# Clindamycin-resistant *Streptococcus pneumoniae*

**DOI:** 10.3201/eid1305.060699

**Published:** 2007-05

**Authors:** Francesca Montagnani, Alessandra Zanchi, Lucia Stolzuoli, Leonardo Croci, Carla Cellesi

**Affiliations:** * Clinica e Laboratorio di Malattie Infettive, Università degli Studi di Siena, Italy; 1 Contributed equally to this work.

**Keywords:** Streptococcus pneumoniae, lincosamide resistance, clindamycin resistance, new phenotype, letter

**To the Editor:** Antimicrobial medications classified as macrolides (e.g., erythromycin) and lincosamides (e.g., clindamycin) show strong activity against streptococci and are commonly used to treat community-acquired infections caused by *Streptococcus pneumoniae*. Moreover, these drugs are the recommended alternatives for patients who cannot tolerate β-lactams.

Two main macrolide-resistant *S. pneumoniae* phenotypes have been reported (*1*). The first has a high level of resistance to all macrolides, lincosamides, ketolides, and streptogramins B due to ribosomal dimethylation, 23S rRNA mutations, or ribosomal protein mutations (MLS_B_, MS_B_, ML, MKS_B_, and K phenotypes). The second is characterized by a low-level resistance (e.g., MIC 2–4 mg/L) to only 14- and 15-member ring macrolides (M phenotype) because of *mef* gene–mediated active drug efflux mechanism.

In January 2005, an erythromycin-susceptible but clindamycin-resistant pneumococcal strain was obtained from a conjunctival swab of a 10-month-old female outpatient attending the daycare center of the Clinic and Laboratory of Infectious Diseases, Siena University, Siena, Italy. To our knowledge, such a phenotype has not been reported in the international literature for *S. pneumoniae*, although a similar phenotype of *Streptococcus agalactiae* was described by Malbruny et al. (*2*).

The *S. pneumoniae* isolate was identified by standard procedures (*3*) and confirmed by PCR for the common capsule gene *cpsA* (*4*). Serotyping, performed by Quellung reaction, showed a 35F serotype. Susceptibility testing was carried out by disk diffusion and confirmed with E-test according to Clinical and Laboratory Standards Institute standards (*5*,*6*) for penicillin, ceftriaxone, ciprofloxacin, erythromycin, clindamycin, linezolid, and quinupristin-dalfopristin. For telithromycin, because an E-test strip was unavailable, a microbroth diluiton method was used.

The strain was susceptible to ceftriaxone (MIC 0.125 mg/L), ciprofloxacin (MIC 0.125 mg/L), erythromycin (MIC 0.125 mg/L), linezolid (MIC 1.5 mg/L), quinupristin/dalfopristin (MIC 0.5 mg/L), and telithromycin (MIC <0.0035 mg/L); it was not susceptible to penicillin (MIC 0.125 mg/L) and was resistant to clindamycin (MIC 1 mg/L). A triple disk–diffusion test with erythromycin, clindamycin, and josamycin was performed to test resistance inducibility. No inducible pattern was shown.

To understand the possible resistance mechanism, MICs for 2 lincosamides (clindamycin and lincomycin) were determined by using a microbroth dilution method in the presence and absence of 10 mg/L of the efflux pump inhibitor reserpine (Sigma Chemicals, St Louis, MO, USA), as described (*7*); *S*. *pneumoniae* ATCC 49619 and *S*. *mitis* 21A29 (*mefE*^+^) were used as controls (*8*). The MICs remained unchanged in the presence of reserpine: 1 mg/L for clindamycin and 4 mg/L for lincomycin.

The strain was screened for *erm*TR, *erm*B or *mef*A, and *mef*E determinants as described (*8*,*9*). All PCR controls gave the expected results. No PCR product was obtained for the studied isolate.

Preliminary data did not show classic macrolide resistance determinants for *S. pneumoniae*. Low-level lincosamide resistance suggests the presence of some efflux mechanism, even if no inhibition by reserpine was observed. Moreover, no mutations of ribosomal proteins and of known binding sites for lincosamides in rRNA (*1*) were shown by sequencing of L22, L4, and 23S rRNA domain II and V genes with primers described by Canu et al. (*10*). Although these findings are preliminary and the molecular basis for resistance is the subject of ongoing investigation, the identification of this *S. pneumoniae* phenotype may affect clinical management of pneumococcal infections, especially in the treatment of patients intolerant of β-lactams.
